# Income-Related Disparities in Mortality Among Young Adults With Type 2 Diabetes

**DOI:** 10.1001/jamanetworkopen.2024.43918

**Published:** 2024-11-12

**Authors:** Ji Yoon Kim, Sojeong Park, Minae Park, Nam Hoon Kim, Sin Gon Kim

**Affiliations:** 1 Department of Internal Medicine, Korea University College of Medicine, Seoul, Republic of Korea; 2 Department of Medicine, Samsung Medical Center, Sungkyunkwan University School of Medicine, Seoul, Republic of Korea; 3 Department of Data Science, Hanmi Pharmaceutical Co Ltd, Seoul, Republic of Korea

## Abstract

**Importance:**

Previous studies have indicated an inverse association between income and mortality. However, differences in health outcomes according to the income level of young adults with type 2 diabetes (T2D) compared with older adults with T2D have not been elucidated.

**Objective:**

To estimate the overall and cause-specific mortality risks among patients with T2D according to income and age.

**Design, Setting, and Participants:**

This retrospective nationwide cohort study in South Korea included adults aged 20 to 79 years who were diagnosed with T2D between January 1, 2008, and December 31, 2013, and followed up until December 31, 2019, and age- and sex-matched controls without diabetes. Data were analyzed between January 1, 2023, and August 27, 2024.

**Main Outcomes and Measures:**

Risks of all-cause, cardiovascular, and cancer mortality were estimated according to participants’ income, which was categorized into 3 levels (low, middle, and high) based on the health insurance premium. Logistic regression analyses and Cox proportional hazard regression analyses were performed according to age groups (20-39, 40-59, and 60-79 years).

**Results:**

A total of 1 240 780 adults (604 975 patients with T2D and 635 805 age- and sex-matched controls without diabetes) were included in the analyses. Their mean (SD) age was 56.9 (11.8) years, and 626 176 (50.5%) were men. Overall, the risk of mortality increased with lower income among patients with T2D, as well as in comparison with controls without diabetes. There was an inverse association between income and mortality risk in younger individuals (adjusted hazard ratios of all-cause mortality in the low income vs high income subgroups with T2D were 2.88 [95% CI, 2.25-3.69] in those aged 20 to 39 years, 1.90 [95% CI, 1.81-2.00] in those aged 40 to 59 years, and 1.26 [95% CI, 1.23-1.29] in those aged 60 to 79 years; *P* < .001 for comparing risk ratios between age groups). The pattern of income-related disparities in younger individuals was observed in cardiovascular mortality but less in cancer mortality.

**Conclusions and Relevance:**

In this cohort study of 1 240 780 individuals aged 20 to 79 years, the risk of mortality with low income was most prominent among individuals with T2D aged 20 to 39 years. These findings highlight the need for socioeconomic support to reduce income-related health disparities in younger individuals.

## Key Points

**Question:**

Are there differences in health outcomes between young adults and older adults with type 2 diabetes (T2D) based on their socioeconomic status?

**Findings:**

In this nationwide cohort study including 1 240 780 adults in South Korea, low income was associated with higher all-cause mortality compared with high income among patients aged 20 to 39 years with T2D, and while still higher, the corresponding values for older groups (those aged 40-59 and those aged 60-79 years) were not as pronounced.

**Meaning:**

These findings suggest that more efforts at the societal level should be made to reduce health disparities among younger patients with T2D.

## Teaser

This cohort study assesses the risk of all-cause and cause-specific mortality according to the income level of adults with type 2 diabetes and whether health outcomes according to income vary by age.

## Introduction

Income is an important socioeconomic indicator that has effects on personal health.^1^ Studies have consistently indicated an inverse association between income and morbidity or mortality in patients with diabetes.^2,3,4,5,6,7^ Low income is associated with a high risk of poor glycemic control,^8^ complications,^9,10,11^ and poor regulation of cardiovascular risk factors.^12,13,14,15^

The incidence and prevalence of type 2 diabetes (T2D) in young individuals is increasing worldwide.^16,17,18^ Patients with early-onset T2D have poor glycemic control and a high risk of diabetic complications. While this is partly due to the biological characteristics of early-onset T2D, it is also influenced by behavioral factors.^19^ Therefore, the effect of socioeconomic status on health outcomes may be more pronounced in these individuals. However, differences in health outcomes according to the socioeconomic status of young adults with T2D compared with older adults with T2D have not been extensively studied. Comparing income-related disparities between young adults and older adults can help determine whether additional interventions are needed to reduce disparities in young adults with T2D.

This cohort study assessed the risk of all-cause and cause-specific mortality according to the income level of adults with T2D. This study also evaluated whether health outcomes according to income varied by age.

## Methods

### Data Source

We used the Korean National Health Insurance Service (NHIS) customized database for January 1, 2008, through December 31, 2019. This database constitutes a nationwide cohort in South Korea covering almost the entire Korean population.^20^ The database contains longitudinal information on the demographic characteristics, diseases, hospitalizations, medical and surgical procedures, prescribed medications, and the results of health examinations of registrants. The database also includes death records. The details of the cohort were described previously.^20,21,22,23^ This study was approved by the Institutional Review Board of Korea University Anam Hospital, Seoul, South Korea. The NHIS cohort data do not include personally identifiable information; thus, the NHIS waived the need for informed consent. We followed the Strengthening the Reporting of Observational Studies in Epidemiology (STROBE) reporting guideline. Data were analyzed from January 1, 2023, to August 27, 2024.

### Study Population

We selected patients diagnosed with T2D, aged 20 to 79 years (N = 965 404), and age- and sex-matched controls without diabetes (N = 1 116 485) between January 1, 2008, and December 31, 2013. Among them, patients who did not undergo a health examination and for whom information on anthropometric and laboratory measurements was not available (360 429 patients with T2D and 480 680 controls without diabetes) were excluded. A total of 1 240 780 adults aged 20 to 79 years (604 975 patients with T2D and 635 805 controls without diabetes) were included in this study.

The index date for all patients with T2D was the first date of documented diagnosis of T2D between January 1, 2008, and December 31, 2013. Patients in the control group without diabetes were matched by age and sex on the index date for the patients with T2D. All patients were followed up from the index date to the end of the cohort period (December 31, 2019) or death, whichever occurred earlier. The median follow-up period was 9.4 (IQR, 7.2-10.6) years.

### Identification of Diabetes, Income, and Mortality

We defined T2D based on the *International Statistical Classification of Diseases, Tenth Revision* (*ICD-10*), codes E11 through E14. Age- and sex-matched controls without diabetes were defined as those who were not clinically diagnosed with diabetes and were not taking any antidiabetic agents.

Individuals’ income level was defined by the medical insurance premium, which was directly proportional to income. The medical insurance premium was originally classified into 20 strata, and we categorized patients’ income into 3 groups (lowest 30%, middle 40%, and highest 30%).

Death due to any cause, cardiovascular causes, or cancer was assessed. Death due to cardiovascular causes was identified based on *ICD-10* codes I00 to I99, and death due to cancer was identified using *ICD-10* codes C00 to C97.

### Statistical Analyses

The participants were categorized into controls without diabetes, patients with T2D and high income, patients with T2D and middle income, and patients with T2D and low income. Mortality risk was estimated based on these groupings. Two sets of analyses were performed: (1) including controls without diabetes as the reference group and (2) including only patients with T2D, with the high-income subgroup as the reference group. The proportional hazards assumption was met within T2D subgroups, but not when including controls without diabetes. Therefore, we conducted logistic regression analyses for the first set and Cox proportional hazard regression analyses for the second set.

The analyses were adjusted for confounding variables, including age, sex, body mass index, systolic blood pressure, current smoking status, alcohol consumption, exercise, underlying atherosclerotic cardiovascular disease (ASCVD), underlying hypertension with antihypertensive treatment, underlying dyslipidemia with lipid-lowering drug treatment, use of antidiabetic medication or not, fasting blood glucose level, low-density lipoprotein cholesterol level, and estimated glomerular filtration rate. There was a significant interaction between groups and age, as well as between groups and other variables in Table 1, except for chronic kidney disease and the use of antidiabetic drugs, regarding all-cause mortality risk.

**Table 1.** Baseline Characteristics of Study Participants^a^

Characteristic	Controls without diabetes (n = 635 805)	Patients with T2D by income subgroup			*P* value	
		High (n = 246 271)	Middle (n = 203 506)	Low (n = 155 198)	Within total population	Within T2D subgroups
Age, mean (SD), y^b^	57.1 (11.9)	58.0 (11.5)	55.3 (11.8)	56.1 (11.4)	<.001	<.001
Sex, No. (%)^c^						
Men	322 358 (50.7)	129 345 (52.5)	103 742 (51.0)	70 731 (45.6)	<.001	<.001
Women	313 447 (49.3)	116 926 (47.5)	99 764 (49.0)	84 467 (54.4)		
BMI, mean (SD)^b^	23.5 (2.9)	24.6 (3.2)	24.6 (3.4)	24.6 (3.5)	<.001	<.001
Waist circumference, mean (SD), cm^b^	80.3 (8.5)	83.7 (9.2)	83.3 (9.1)	83.0 (9.4)	<.001	<.001
SBP, mean (SD), mm Hg^b^	124.0 (15.4)	126.6 (15.4)	126.7 (15.8)	126.5 (15.9)	<.001	.002
Current smoking, No. (%)^c^	126 936 (20.0)	42 354 (17.2)	45 216 (22.2)	32 752 (21.1)	<.001	<.001
Alcohol consumption, No. (%)^c^^,^^d^						
None	380 054 (59.8)	150 317 (61.0)	121 986 (59.9)	98 668 (63.6)	<.001	<.001
≤2 times/wk	206 768 (32.5)	76 370 (31.0)	63 490 (31.2)	44 205 (28.5)		
≥3 times/wk	44 422 (7.0)	17 922 (7.3)	16 847 (8.3)	11 510 (7.4)		
Regular exercise, No. (%)^c^^,^^d^						
None	165 632 (26.1)	59 157 (24.0)	57 365 (28.2)	45 154 (29.1)	<.001	<.001
≤2 times/wk	218 867 (34.4)	87 529 (35.5)	67 852 (33.3)	49 213 (31.7)		
≥3 times/wk	248 845 (39.1)	98 761 (40.1)	77 686 (38.2)	60 375 (38.9)		
Comorbidity, No. (%)^c^						
Hypertension	188 487 (29.6)	140 473 (57.0)	106 738 (52.4)	84 062 (54.2)	<.001	<.001
Dyslipidemia	185 115 (29.1)	149 529 (60.7)	111 223 (54.7)	85 998 (55.4)	<.001	<.001
ASCVD	10 928 (1.7)	13 569 (5.5)	9304 (4.6)	7188 (4.6)	<.001	<.001
Ischemic heart disease	5712 (0.9)	8695 (3.5)	5611 (2.8)	4109 (2.6)	<.001	<.001
Stroke	5403 (0.8)	5327 (2.2)	3946 (1.9)	3308 (2.1)	<.001	<.001
Chronic kidney disease^e^	89 275 (14.0)	37 656 (15.3)	26 170 (12.9)	21 569 (13.9)	<.001	<.001
Cancer	44 823 (7.0)	31 105 (12.6)	20 752 (10.2)	16 069 (10.4)	<.001	<.001
Laboratory findings, mean (SD)^b^						
FBG level, mg/dL	93.8 (12.4)	111.6 (34.3)	113.0 (38.7)	112.2 (38.2)	<.001	<.001
TC level, mg/dL	199.5 (38.5)	196.1 (42.4)	197.0 (42.6)	197.0 (44.5)	<.001	<.001
LDL-C level, mg/dL	120.4 (85.6)	115.5 (81.1)	115.1 (83.0)	114.7 (65.5)	<.001	.01
HDL-C level, mg/dL	56.1 (30.4)	53.5 (25.1)	53.8 (24.5)	54.4 (35.4)	<.001	<.001
Triglyceride level, mg/dL	127.9 (82.3)	148.4 (100.4)	152.9 (111.0)	150.1 (107.5)	<.001	<.001
eGFR, mL/min/1.73m^2^	81.7 (27.4)	82.1 (31.0)	84.8 (31.0)	83.6 (30.6)	<.001	<.001
Use of antidiabetic drugs, No. (%)^c^						
Metformin	0	33 637 (13.7)	26 500 (13.0)	19 160 (12.3)	<.001	<.001
Sulfonylurea	0	39 260 (15.9)	31 360 (15.4)	22 882 (14.7)	<.001	<.001
AGI	0	13 329 (5.4)	10 689 (5.3)	7983 (5.1)	<.001	.001
Glinide	0	3396 (1.4)	2509 (1.2)	1819 (1.2)	<.001	<.001
Thiazolidinedione	0	3885 (1.6)	2584 (1.3)	1702 (1.1)	<.001	<.001
DPP-4 inhibitor	0	298 (0.1)	260 (0.1)	230 (0.1)	<.001	.06
SGLT2 inhibitor	0	0	0	0	NA	NA
GLP1RA	0	0	0	0	NA	NA
Insulin	0	9690 (3.9)	7421 (3.6)	5740 (3.7)	<.001	<.001
Use of antihypertensives, No. (%)^c^						
RAS inhibitor	97 953 (15.4)	87 355 (35.5)	66 397 (32.6)	52 214 (33.6)	<.001	<.001
Calcium channel blocker	132 604 (20.9)	100 942 (41.0)	78 163 (38.4)	61 774 (39.8)	<.001	<.001
β-Blocker	70 455 (11.1)	61 309 (24.9)	47 307 (23.2)	37 590 (24.2)	<.001	<.001
Diuretics	139 274 (21.9)	96 637 (39.2)	77 359 (38.0)	62 028 (40.0)	<.001	<.001
α-Blocker or vasodilator	22 598 (3.6)	15 972 (6.5)	10 730 (5.3)	8311 (5.4)	<.001	<.001
Use of lipid-lowering drugs, No. (%)^c^						
Statin	81 364 (12.8)	82 502 (33.5)	59 903 (29.4)	46 761 (30.1)	<.001	<.001
Fibrate	10 732 (1.7)	17 200 (7.0)	12 380 (6.1)	9129 (5.9)	<.001	<.001
Use of antithrombotics, No. (%)^c^	102 204 (16.1)	88 609 (36.0)	63 996 (31.4)	50 336 (32.4)	<.001	<.001
No. of clinic visits per year, mean (SD)^b^	16.6 (15.8)	26.5 (21.2)	26.0 (21.8)	28.1 (23.1)	<.001	<.001
No. of times participating in the national health checkup program, mean (SD)^b^	4.6 (2.6)	4.6 (2.5)	4.3 (2.4)	4.1 (2.5)	<.001	<.001

Abbreviations: AGI, α-glucosidase inhibitor; ASCVD, atherosclerotic cardiovascular disease; BMI, body mass index (calculated as the weight in kilograms divided by the height in meters squared); DPP-4, dipeptidyl peptidase-4; eGFR, estimated glomerular filtration rate; FBG, fasting blood glucose; GLP1RA, glucagonlike peptide-1 receptor agonist; HDL-C, high-density lipoprotein cholesterol; LDL-C, low-density lipoprotein cholesterol; RAS, renin-angiotensin system; SBP, systolic blood pressure; SGLT2, sodium-glucose cotransporter 2; T2D, type 2 diabetes; TC, total cholesterol.

SI conversion factors: To convert FBG to mmol/L, multiply by 0.0555; HDL-C, LDL-C, and TC to mmol/L, multiply by 0.0259; triglyceride to mmol/L, multiply by 0.0113.

^a^ Owing to missing data for some characteristics, percentages may not total 100.

^b^ Compared using 1-way analysis of variance.

^c^ Compared using Pearson χ^2^ test or Fisher exact test.

^d^ The Cochran-Mantel-Haenszel test was also conducted to investigate the trend relation between the ordinal factors and ordinal T2D groups; *P* < .001 for trend for both variables.

^e^ Defined as eGFR of less than 60 mL/min/1.73 m^2^.

Following the analyses in the total population and the T2D subgroups aged 20 to 79 years, additional analyses were performed within individual age groups (20-39, 40-59, and 60-79 years). The same logistic regression model and Cox proportional hazard regression model were used within each age group. We also compared the mortality risk ratio of the T2D subgroups with low vs high income among patients aged 20 to 39 years and those aged 60 to 79 years.

Next, we examined the incidence of ASCVD and cancer according to income status. ASCVD was classified as either ischemic heart disease (IHD) or stroke. IHD was defined as hospitalizations for IHD (*ICD-10* codes I20-I25) with records of coronary artery angiography or procedures. Stroke events were defined as hospitalizations for stroke (*ICD-10* codes I60-64) with brain imaging. Cancer was identified based on *ICD-10* codes C00 to C97. If the participant had a history of ASCVD or cancer before the index date, they were classified as having these comorbidities at baseline. Analyses of the incidence of ASCVD and cancer were conducted among those without a history of ASCVD or cancer at baseline, respectively.

All the statistical analyses were performed using SAS, version 9.4 (SAS Institute Inc). Statistical significance was set at a 2-sided *P* < .05.

## Results

### Baseline Characteristics

Table 1 presents the baseline characteristics of the 1 240 780 study participants. Overall mean (SD) age was 56.9 (11.8) years and ranged from 55.3 (11.8) to 58.0 (11.5) years in the income subgroups. A total of 626 176 participants (50.5%) were men and 614 604 (49.5%) were women. The proportion of men was lower in the T2D subgroup with low income (70 731 of 155 198 [45.6%]) than in the T2D subgroups with middle (103 742 of 203 506 [51.0%]) and high income (129 345 of 246 271 [52.5%]). The proportion of current smokers was lowest in the T2D subgroup with high income. For alcohol consumption and exercise in T2D groups, there was a trend between the ordinal factors and ordinal T2D groups. While the proportion of participants who did not consume alcohol was highest in the T2D subgroup with low income, the proportion of participants who did not exercise regularly was lowest in the T2D subgroup with high income. The proportion of participants with hypertension, dyslipidemia, ASCVD, and a history of cancer was higher among patients with T2D than in the controls without diabetes. Among the patients with T2D, 30 061 (5.0%) had ASCVD, and 67 926 (11.2%) had a history of cancer at baseline. The use of antidiabetic drugs (except for dipeptidyl peptidase-4 inhibitors), antihypertensives, and lipid-lowering drugs was more prevalent in the T2D subgroup with high income than in the T2D subgroups with middle and low income. In contrast, the number of clinic visits was highest in the T2D subgroup with low income. Baseline characteristics of the participants according to age groups are presented in the eTable 1 in Supplement 1. The characteristics showed similar findings with those of the total population; however, the mean number of participations in the national health checkup program was lowest in the T2D subgroup with low income, particularly among younger (20-39 and 40-59 years) groups.

### Risk of Mortality According to Income

Compared with the controls without diabetes, the adjusted odds ratios of all-cause mortality were 1.47 (95% CI, 1.44-1.50) in the T2D subgroup with high income, 1.79 (95% CI, 1.75-1.83) in the T2D subgroup with middle income, and 2.03 (95% CI, 1.99-2.08) in the T2D subgroup with low income (Table 2). Among T2D group, adjusted hazard ratios (AHRs) for all-cause mortality were 1.19 (95% CI, 1.17-1.22) in those with middle income and 1.35 (95% CI, 1.32-1.38) in those with low income with the high-income subgroup as reference. Similar patterns were observed in both men and women (eTable 2 in Supplement 1).

**Table 2.** Risk of Overall and Cause-Specific Mortality According to Income

Participant group	No. of deaths/No. of participants (%)	Event rate per 1000 person-years	OR (95% CI)^a^		HR (95% CI)^b^	
			Unadjusted	Adjusted^c^	Unadjusted	Adjusted^c^
**All-cause mortality**						
Controls without diabetes	31 665/635 805 (5.0)	5.83	1 [Reference]	1 [Reference]	NA	NA
T2D subgroup						
High income	19 710/246 271 (8.0)	8.56	1.66 (1.63-1.69)	1.47 (1.44-1.50)	1 [Reference]	1 [Reference]
Middle income	15 883/203 506 (7.8)	8.43	1.62 (1.58-1.65)	1.79 (1.75-1.83)	0.99 (0.97-1.01)	1.19 (1.17-1.22)
Low income	13 753/155 198 (8.9)	9.68	1.86 (1.82-1.89)	2.03 (1.99-2.08)	1.14 (1.12-1.17)	1.35 (1.32-1.38)
**Death due to cardiovascular disease**						
Controls without diabetes	5623/635 805 (0.9)	1.03	1 [Reference]	1 [Reference]	NA	NA
T2D subgroup						
High income	3938/246 271 (1.6)	1.71	1.82 (1.75-1.90)	1.32 (1.26-1.38)	1 [Reference]	1 [Reference]
Middle income	3115/203 506 (1.5)	1.65	1.74 (1.67-1.82)	1.60 (1.53-1.68)	0.97 (0.93-1.02)	1.23 (1.17-1.29)
Low income	2726/155 198 (1.8)	1.92	2.00 (1.91-2.10)	1.80 (1.71-1.89)	1.14 (1.08-1.19)	1.40 (1.33-1.47)
**Death due to cancer**						
Controls without diabetes	12 625/635 805 (2.0)	2.32	1 [Reference]	1 [Reference]	NA	NA
T2D subgroup						
High income	7495/246 271 (3.0)	3.26	1.55 (1.51-1.60)	1.47 (1.42-1.51)	1 [Reference]	1 [Reference]
Middle income	5881/203 506 (2.9)	3.12	1.47 (1.42-1.52)	1.68 (1.63-1.74)	0.96 (0.93-0.99)	1.15 (1.11-1.19)
Low income	4864/155 198 (3.1)	3.42	1.60 (1.54-1.65)	1.80 (1.73-1.86)	1.06 (1.02-1.10)	1.24 (1.20-1.29)

Abbreviations: HR, hazard ratio; NA, not applicable; OR, odds ratio; T2D, type 2 diabetes.

^a^ Within the total population, including controls without diabetes and individuals with T2D.

^b^ Within individuals with T2D.

^c^ Adjusted for age, sex, body mass index, systolic blood pressure, current smoking, drinking, exercise, underlying atherosclerotic cardiovascular disease, underlying hypertension treated with antihypertensives, underlying dyslipidemia treated with lipid-lowering drugs, use of antidiabetic drugs, fasting blood glucose level, low-density lipoprotein cholesterol level, and estimated glomerular filtration rate.

A similar association was observed for cause-specific mortality. Compared with the T2D subgroup with high income, cardiovascular mortality risk was higher among the T2D subgroup with middle income (AHR, 1.23 [95% CI, 1.17-1.29]) and among the T2D subgroup with low income (AHR, 1.40 [95% CI, 1.33-1.47]). Cancer mortality risk was higher in the T2D subgroup with middle income (AHR, 1.15 [95% CI, 1.11-1.19]) and in the T2D subgroup with low income (AHR, 1.24 [95% CI, 1.20-1.29]) (Table 2).

### Risk of Mortality According to Income and Age

**Figure 1.**
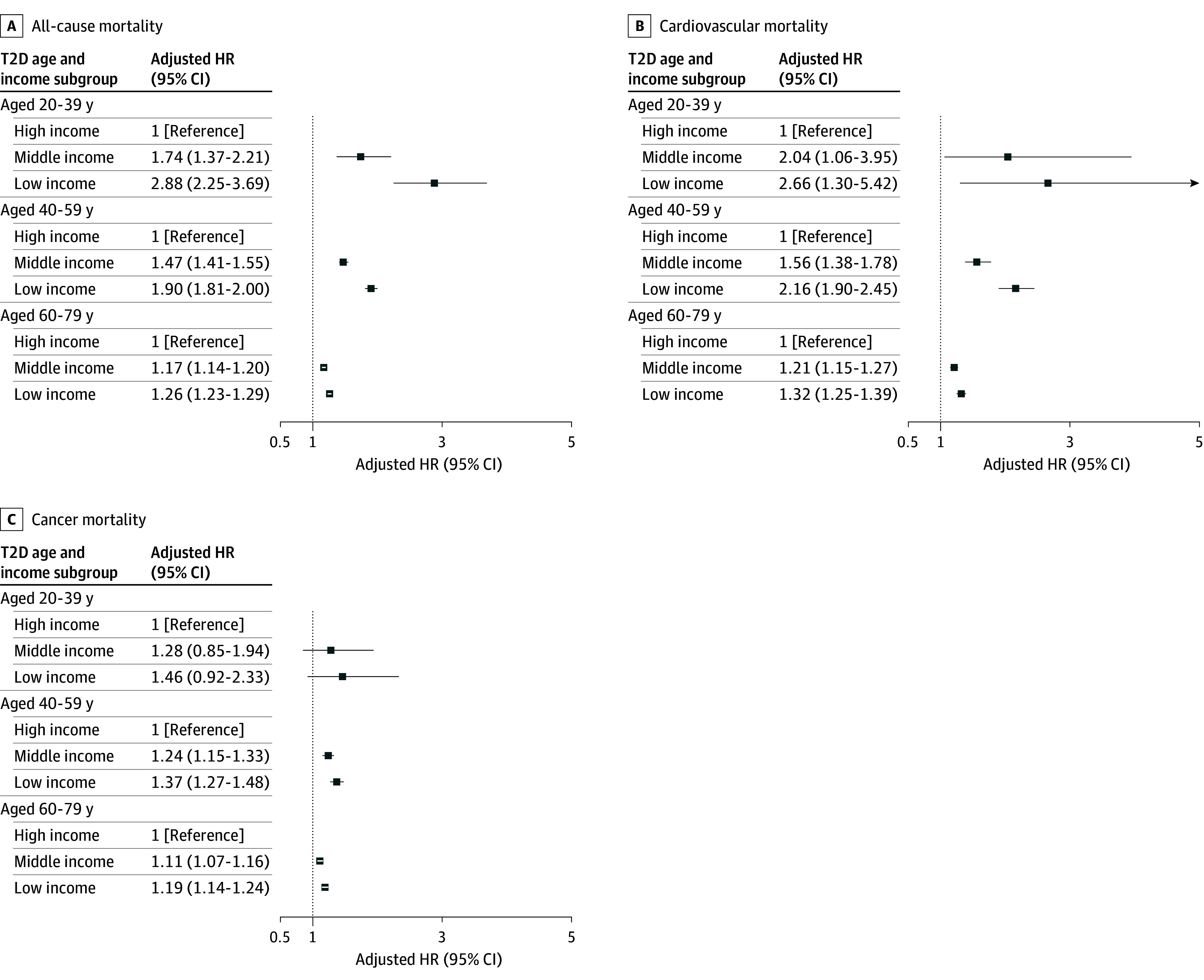
Mortality Risk by Income and Age Among Individuals With Type 2 Diabetes (T2D) Hazard ratios (HRs) are adjusted for age, sex, body mass index, systolic blood pressure, current smoking, drinking, exercise, underlying atherosclerotic cardiovascular disease, underlying hypertension treated with antihypertensives, underlying dyslipidemia treated with lipid-lowering drugs, use of antidiabetic drugs, fasting blood glucose level, low-density lipoprotein cholesterol level, and estimated glomerular filtration rate.

The inverse association between income and mortality was consistently observed across all age groups (20-39, 40-59, and 60-79 years) (eFigure in Supplement 1), but it was more prominent in younger adults than in older adults (Table 3 and Figure 1). Compared with the controls without diabetes, the adjusted odds ratios of all-cause mortality in the T2D subgroup with low income were 4.05 (95% CI, 3.29-5.00) in people aged 20 to 39 years, 2.76 (95% CI, 2.63-2.89) in those aged 40 to 59 years, and 1.82 (95% CI, 1.77-1.87) in those aged 60 to 79 years. Among patients with T2D, the AHRs of all-cause mortality in the T2D subgroup with low income were 2.88 (95% CI, 2.25-3.69) in those aged 20 to 39 years, 1.90 (95% CI, 1.81-2.00) in those aged 40 to 59 years, and 1.26 (95% CI, 1.23-1.29) in those aged 60 to 79 years compared with the T2D subgroup with high income. There was a significant interaction between age groups and income status (*P* < .001 for interaction). As shown, the risk ratio of the T2D subgroups with low vs high income among those aged 20 to 39 years was greater than that among patients aged 60 to 79 years (*P* < .001).

**Table 3.** Risk of Overall and Cause-Specific Mortality According to Income and Age

Participant group	Aged 20-39 y				Aged 40-59 y				Aged 60-79 y			
	No. of events/No. of participants (%)	Event rate per 1000 person-years	Adjusted risk		No. of events/No. of participants (%)	Event rate per 1000 person-years	Adjusted		No. of events/No. of participants (%)	Event rate per 1000 person-years	Adjusted	
			OR (95% CI)^a^^,^^b^	HR (95% CI)^b^^,^^c^			OR (95% CI)^a^^,^^b^	HR (95% CI)^b^^,^^c^			OR (95% CI)^a^^,^^b^	HR (95% CI)^b^^,^^c^
**Death due to any cause**												
Controls without diabetes	193/48 457 (0.4)	0.44	1 [Reference]	NA	4655/294 858 (1.6)	1.81	1 [Reference]	NA	26 817/292 490 (9.2)	11.05	1 [Reference]	NA
T2D subgroup												
High income	96/14 129 (0.7)	0.72	1.40 (1.08-1.80)	1 [Reference]	2912/117 206 (2.5)	2.62	1.44 (1.37-1.51)	1 [Reference]	16 702/114 936 (14.5)	15.81	1.43 (1.40-1.47)	1 [Reference]
Middle income	242/22 027 (1.1)	1.19	2.39 (1.96-2.92)	1.74 (1.37-2.21)	3931/103 024 (3.8)	4.07	2.12 (2.03-2.23)	1.47 (1.41-1.55)	11 710/78 455 (14.9)	16.38	1.70 (1.65-1.74)	1.17 (1.14-1.20)
Low income^d^^,^^e^	206/11 941 (1.7)	1.86	4.05 (3.29-5.00)	2.88 (2.25-3.69)	3843/80 948 (4.7)	5.12	2.76 (2.63-2.89)	1.90 (1.81-2.00)	9704/62 309 (15.6)	17.38	1.82 (1.77-1.87)	1.26 (1.23-1.29)
**Death due to cardiovascular disease**												
Controls without diabetes	26/48 457 (0.1)	0.06	1 [Reference]	NA	628/294 858 (0.2)	0.24	1 [Reference]	NA	4969/292 490 (1.7)	2.05	1 [Reference]	NA
T2D subgroup												
High income	12/14 129 (0.1)	0.09	0.90 (0.44-1.84)	1 [Reference]	400/117 206 (0.3)	0.36	1.19 (1.04-1.36)	1 [Reference]	3526/114 936 (3.1)	3.34	1.31 (1.25-1.38)	1 [Reference]
Middle income	37/22 027 (0.2)	0.18	1.90 (1.10-3.28)	2.04 (1.06-3.95)	583/103 024 (0.6)	0.60	1.83 (1.62-2.08)	1.56 (1.38-1.78)	2495/78 455 (3.2)	3.49	1.57 (1.49-1.66)	1.21 (1.15-1.27)
Low income^f^^,^^g^	23/11 941 (0.2)	0.21	2.49 (1.36-4.56)	2.66 (1.30-5.42)	621/80 948 (0.8)	0.83	2.51 (2.22-2.84)	2.16 (1.90-2.45)	2082/62 309 (3.3)	3.73	1.67 (1.58-1.77)	1.32 (1.25-1.39)
**Death due to cancer**												
Controls without diabetes	32/48 457 (0.1)	0.07	1 [Reference]	NA	1856/294 858 (0.6)	0.72	1 [Reference]	NA	10 737/292 490 (3.7)	4.43	1 [Reference]	NA
T2D subgroup												
High income	36/14 129 (0.3)	0.27	3.21 (1.96-5.26)	1 [Reference]	1407/117 206 (1.2)	1.27	1.90 (1.77-2.05)	1 [Reference]	6052/114 936 (5.3)	5.73	1.68 (1.34-1.44)	1 [Reference]
Middle income	64/22 027 (0.3)	0.32	4.07 (2.62-6.32)	1.28 (0.85-1.94)	1576/103 024 (1.5)	1.63	2.32 (2.16-2.50)	1.24 (1.15-1.33)	4241/78 455 (5.4)	5.93	1.53 (1.47-1.59)	1.11 (1.07-1.16)
Low income^d^^,^^h^	39/11 941 (0.3)	0.35	4.59 (2.82-7.48)	1.46 (0.92-2.33)	1317/80 948 (1.6)	1.75	2.53 (2.34-2.73)	1.37 (1.27-1.48)	3508/62 309 (5.6)	6.28	1.62 (1.55-1.69)	1.19 (1.14-1.24)

Abbreviations: HR, hazard ratio; NA, not applicable; OR, odds ratio; T2D, type 2 diabetes.

^a^ Calculated from models fitted in individual age ranges, including controls without diabetes and individuals with T2D.

^b^ Adjusted for age, sex, body mass index, systolic blood pressure, current smoking, drinking, exercise, underlying atherosclerotic cardiovascular disease, underlying hypertension treated with antihypertensives, underlying dyslipidemia treated with lipid-lowering drugs, use of antidiabetic drugs, fasting blood glucose level, low-density lipoprotein cholesterol level, and estimated glomerular filtration rate.

^c^ Within individuals with T2D. HRs were calculated from the models fitted in individual age ranges.

^d^ *P* < .001 comparing the OR of the T2D subgroup with low income vs controls without diabetes in individuals aged 20 to 39 years and 60 to 79 years.

^e^ *P* < .001 comparing the HR of the T2D subgroups with low income vs high income in individuals aged 20 to 39 years and 60 to 79 years.

^f^ *P* = .007 comparing the OR of the T2D subgroup with low income vs controls without diabetes in individuals aged 20 to 39 years and 60 to 79 years.

^g^ *P* = .04 comparing the HR of the T2D subgroups with low income vs high income in individuals aged 20 to 39 years and 60 to 79 years.

^h^ *P* = .23 comparing the HR of the T2D subgroups with low income vs high income in individuals aged 20 to 39 years and 60 to 79 years.

The cardiovascular mortality risk according to income and age showed similar patterns to the risk for all-cause mortality. In patients aged 20 to 39 years, the AHR of the T2D subgroup with low income was 2.66 (95% CI, 1.30-5.42) compared with the T2D subgroup with high income and 1.32 (95% CI, 1.25-1.39) in patients aged 60 to 79 years. Regarding cancer mortality, in patients aged 20 to 39 years, the AHR of the T2D subgroup with low income compared to the T2D subgroup with high income was 1.46 (95% CI, 0.92-2.33) and was 1.19 (95% CI, 1.14-1.24) in patients aged 60 to 79 years.

### Risk of Incident ASCVD and Cancer According to Income

We investigated whether the risk of developing ASCVD and cancer differed according to the income level (eTable 3 in Supplement 1). Overall, the risks of incident ASCVD and cancer were higher in patients with T2D than in the controls without diabetes.

**Figure 2.**
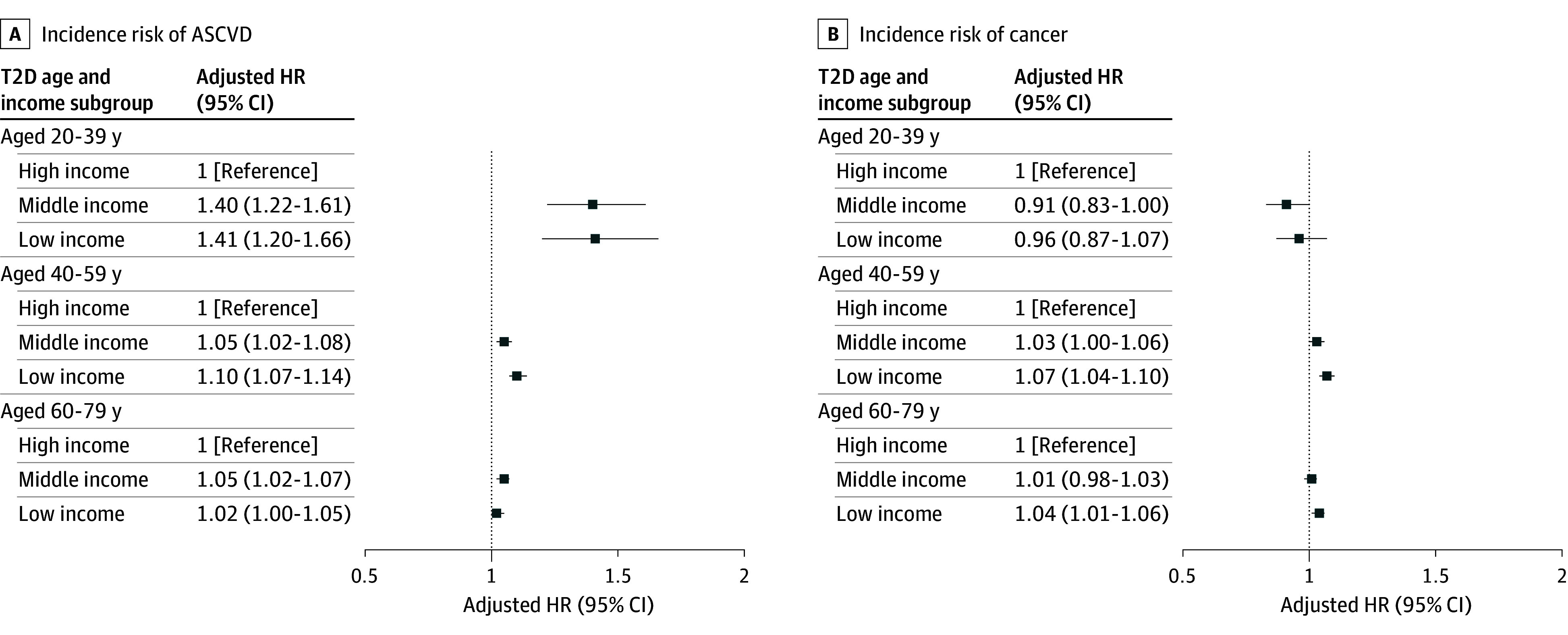
Incidence Risk by Income and Age Among Individuals With Type 2 Diabetes (T2D) Hazard ratios (HRs) are adjusted for age, sex, body mass index, systolic blood pressure, current smoking, drinking, exercise, underlying atherosclerotic cardiovascular disease (ASCVD), underlying hypertension treated with antihypertensives, underlying dyslipidemia treated with lipid-lowering drugs, use of antidiabetic drugs, fasting blood glucose level, low-density lipoprotein cholesterol level, and estimated glomerular filtration rate.

The risks of ASCVD and cancer according to income and age are shown in eTable 4 in Supplement 1 and Figure 2. The inverse association between income and ASCVD incidence was prominent in younger patients with T2D. In patients aged 20 to 39 years, the risk of developing ASCVD was higher in the T2D subgroup with low income than in the T2D subgroup with high income (AHR, 1.41; 95% CI, 1.20-1.66). In contrast, in patients aged 60 to 79 years, the risk of developing ASCVD was similar (AHR, 1.02 [95% CI, 1.00-1.05]) between the T2D subgroup with low income and T2D subgroup with high income. The risk of incident cancer did not differ according to income status in patients aged 20 to 39 years.

## Discussion

In this nationwide cohort study of 1 240 780 individuals, we found that income was a critical factor for determining mortality risk in patients with T2D. Notably, income-related disparities in mortality were prominent in adults younger than 40 years. Among young adults with T2D, low income was associated with an AHR of 2.88 for all-cause mortality compared with the subgroup with high income, while the corresponding values for older patients were smaller (1.90 for patients aged 40-59 years and 1.26 for patients aged 60-79 years). Pronounced income-related disparity among young adults with T2D was observed for cardiovascular mortality but less for cancer mortality. The overall results indicate that diabetes-related management and health policies must focus on younger adult populations with T2D.

The prevalence of T2D is increasing globally, and there is an unequal burden of the disease on people in low- and middle-income countries.^24,25^ Therefore, the Lancet Commission on Diabetes and the World Health Organization Global Diabetes Compact called for multilayered approaches to address these challenges.^26^ In line with this global recommendation, our study also supports the need for additional efforts to reduce income-related disparities. Moreover, our study calls for special attention to young adults with T2D. To the best of our knowledge, this is the first study comparing young adults with T2D and older patients regarding income-related disparities in mortality. Notably, low income was the most powerful factor associated with all-cause mortality among various factors in adults with T2D aged 20 to 39 years (eTable 5 in Supplement 1).

South Korea has implemented health insurance at the national level,^27^ and health insurance premiums were calculated in proportion to individual income. This approach has the advantage of measuring income levels more objectively than other studies that rely on self-reported income.^28,29^ Income influences individuals’ health in multiple ways, including access to health care.^30,31^ South Korea’s nationwide health insurance system ensures minimal discrimination in terms of accessibility to health care for all citizens. In this study, the annual clinical visit rate was not lower in the low-income group than in the high-income group. Therefore, an important highlight of this study is that even where differences in medical accessibility according to socioeconomic status are minimal, income remains an independent risk factor for mortality.

The pronounced income-related disparity in mortality among young adults was probably influenced by a complex interplay of various other factors. The difference in the occurrence of ASCVD by income was greater in patients with T2D who were younger than 40 years than in those 40 years or older, which probably contributes to the greater disparity in cardiovascular mortality among younger people with T2D. Of note, income was associated with mortality risk rather than morbidity. While the AHR for incident ASCVD of the T2D subgroup with low income among patients aged 20 to 39 years was 1.41, the AHR for cardiovascular mortality was 2.66 compared with the T2D subgroup with high income. This suggests that there may be differences in treatment after the occurrence of ASCVD according to socioeconomic status. Even within a system that ensures access to care, a myriad of complex factors might still influence use of health care services. The use of antidiabetic drugs, antihypertensives, and lipid-lowering drugs was more prevalent in the T2D subgroup with high income than in the subgroups with middle and low income, which could potentially affect mortality. Considering that income is closely associated with treatment adherence, this might also affect mortality.^32,33,34^ In particular, treatment adherence in young adults with T2D in South Korea has been reported to be very low.^19^ However, other factors besides the use of medication likely also affected health outcomes. Young individuals with low income participated in the national health checkup program less frequently than those with high income. This suggests that young people with low income might find it difficult to prioritize their health before the onset of disease.

T2D in East Asian individuals has some distinctive features compared with T2D in Western individuals, 1 of which is that the leading cause of death in East Asian patients with T2D is cancer, not ASCVD.^35^ Indeed, the most common cause of death in our study population was malignant neoplasms, followed by cardiovascular disease (eTable 6 in Supplement 1). However, the differences according to income level were more pronounced for cardiovascular mortality than for cancer mortality (HRs for cardiovascular mortality and cancer mortality among the T2D subgroup with low income were 1.40 [95% CI, 1.33-1.47] and 1.24 [95% CI, 1.20-1.29], respectively, compared with the subgroup with high income). Therefore, to narrow the gap in income-related disparities in mortality, thorough management of cardiovascular disease is required in socioeconomically vulnerable populations.

Previous studies^36,37,38^ indicated that sex differences exist in the control of cardiovascular risk factors and absolute cardiovascular risks. In our study, the death rate was higher in men than in women among patients with T2D. However, the income-related disparities in mortality were similar between men and women. This finding suggests that an individual’s income is a universal risk factor for mortality.

### Limitations

This study had several limitations. First, because this study was based on a retrospective cohort database, there might have been residual confounders, although we extensively adjusted for possible confounders in the analysis. Second, multiple socioeconomic indicators interrelatedly affect health outcomes; however, this study did not consider other socioeconomic indicators such as occupation, educational attainment, and marital status, owing to a lack of relevant information. Third, the median follow-up duration of the study subjects was 9.4 years, which is a relatively short time for observing long-term mortality outcomes, specifically for young adults with T2D. Fourth, we did not investigate causes of death other than cardiovascular or cancer mortality, because the number of disease-related deaths, other than those related to cardiovascular disease or cancer, was low in young adults aged 20 to 39 years (eTable 7 in Supplement 1). Due to restrictions on relevant data, we were unable to analyze specific causes of death unrelated to diseases, such as suicide. Fifth, T2D was defined based on *ICD-10* codes E11 to E14; however, codes E12 to E14 include miscellaneous causes of diabetes, such as malnutrition. We conducted additional separate analyses of individuals with T2D based on code E11 and those with T2D based on codes E12 to E14 (eTable 8 in Supplement 1), which showed similar results to the overall analyses. Last, this study was conducted in South Korea, where a nationwide health insurance system covers the entire population, making it difficult to generalize the results to other countries with different health care systems. Given South Korea’s high level of medical accessibility, other countries lacking such conditions might exhibit more serious disparities.

## Conclusions

In this cohort study of 1 240 780 individuals, individual income level was an independent risk factor for mortality in patients with T2D, and the income-related disparity in mortality was pronounced in young people. This provides epidemiologic evidence of the need for health care policies targeting young people with T2D. More efforts at the social and national level should be made to reduce the disparities in health outcomes according to socioeconomic status among young adults with T2D.

## Notes

Supplement 1. **eTable 1.** Baseline Characteristics of Study Participants in Each Age Group **eTable 2.** Risk of All-Cause Mortality According to Sex and Income **eTable 3.** Risk of Incident Atherosclerotic Cardiovascular Diseases and Cancer According to Income **eTable 4.** Risk of Incident Atherosclerotic Cardiovascular Diseases and Cancer According to Income and Age **eTable 5.** Multivariable Analysis of All-Cause Mortality in Individuals With Type 2 Diabetes Aged 20 to 39 Years **eTable 6.** Causes of Death in the Total Population **eTable 7.** Causes of Death by Age and Income **eTable 8.** Analysis of the Risk of Overall and Cause-Specific Mortality by Income and Age, Using Different *ICD-10* Codes for T2D **eFigure.** Mortality Rate According to Income and Age

Supplement 2.
Data Sharing Statement
