# Supplementary material for: Income-Related Disparities in Mortality Among Young Adults With Type 2 Diabetes
Source: JAMA Netw Open. 2024 Nov 12;7(11):e2443918. doi: 10.1001/jamanetworkopen.2024.43918 (PMC11558478; doi:10.1001/jamanetworkopen.2024.43918)
Supplement: Supplement 1. — eTable 1. Baseline Characteristics of Study Participants in Each Age Group eTable 2. Risk of All-Cause Mortality According to Sex and Income eTable 3. Risk of Incident Atherosclerotic Cardiovascular Diseases and Cancer According to Income eTable 4. Risk of Incident Atherosclerotic Cardiovascular Diseases and Cancer According to Income and Age eTable 5. Multivariable Analysis of All-Cause Mortality in Individuals With Type 2 Diabetes Aged 20 to 39 Years eTable 6. Causes of Death in the Total Population eTable 7. Causes of Death by Age and Income eTable 8. Analysis of the Risk of Overall and Cause-Specific Mortality by Income and Age, Using Different ICD-10 Codes for T2D eFigure. Mortality Rate According to Income and Age [file jamanetwopen-e2443918-s001.pdf]

Supplementary Online Content

Kim JY, Park S, Park M, Kim NH, Kim SG. Income-related disparities in mortality among young adults with type 2 diabetes. *JAMA Netw Open*. 2024;7(11):e2443918. doi:10.1001/jamanetworkopen.2024.43918

eTable 1. Baseline Characteristics of Study Participants in Each Age Group

eTable 2. Risk of All-Cause Mortality According to Sex and Income

eTable 3. Risk of Incident Atherosclerotic Cardiovascular Diseases and Cancer According to Income

eTable 4. Risk of Incident Atherosclerotic Cardiovascular Diseases and Cancer According to Income and Age

eTable 5. Multivariable Analysis of All-Cause Mortality in Individuals With Type 2 Diabetes Aged 20 to 39 Years

eTable 6. Causes of Death in the Total Population

eTable 7. Causes of Death by Age and Income

eTable 8. Analysis of the Risk of Overall and Cause-Specific Mortality by Income and Age, Using Different *ICD-10* Codes for T2D

eFigure. Mortality Rate According to Income and Age

This supplementary material has been provided by the authors to give readers additional information about their work.

© 2024 Kim JY et al. *JAMA Network Open*.

eTable 1. Baseline Characteristics of Study Participants in Each Age Group

	Controls without diabetes	Type 2 diabetes High income	Type 2 diabetes Middle income	Type 2 diabetes Low income	p-value[a]	p-value[b]
**(A) 20-39 years**						
**N**	48,457	14,129	22,027	11,941		
**Age (years)[c]**	32.8 (4.7)	34.9 (4.3)	33.2 (4.4)	32.9 (5.2)	<.001	<.001
**Sex, n(%)[d]**					<.001	<.001
Men	31,285 (64.6)	9,377 (66.4)	13,740 (62.4)	5,716 (47.9)		
Women	17,172 (35.4)	4,752 (33.6)	8,287 (37.6)	6,255 (52.1)		
**BMI (kg/m^2)[c]**	23.0 (3.4)	24.7 (4.1)	24.7 (4.4)	24.3 (4.5)	<.001	<.001
**WC (cm)[c]**	78.0 (9.5)	82.5 (11.1)	81.7 (11.5)	80.3 (11.7)	<.001	<.001
**SBP (mmHg)[c]**	118 (13.0)	121.1 (14.6)	121.2 (14.9)	119.8 (14.7)	<.001	<.001
**Current Smoking, n(%)[d]**	17,292 (35.7)	4,716 (33.4)	8,280 (37.6)	3,792 (31.8)	<.001	<.001
**Alcohol consumption, n(%)[d]**					<.001	<.001
None	17,684 (36.5)	5,341 (37.8)	8,716 (39.6)	5,669 (47.5)		
≤2 times/week	28,490 (58.8)	8,064 (57.1)	12,113 (55.0)	5,607 (47.0)		
≥3 times/week	1,868 (3.9)	620 (4.4)	1,110 (5.0)	614 (5.1)		
**Exercise, n(%)[d]**					<.001	<.001
None	9,401 (19.4)	2,448 (17.3)	4,588 (20.8)	2,695 (22.6)		
≤2 times/week	21,266 (43.9)	6,688 (47.3)	9,577 (43.5)	4,825 (40.4)		
≥3 times/week	17,505 (36.1)	4,935 (34.9)	7,791 (35.4)	4,393 (36.8)		
**Comorbidity, n(%)[d]**						
Hypertension	1,837 (3.8)	2,795 (19.8)	3,709 (16.8)	1,954 (16.4)	<.001	<.001
Dyslipidemia	4,673 (9.6)	5,176 (36.6)	6,673 (30.3)	3,584 (30.0)	<.001	<.001
ASCVD	55 (0.1)	104 (0.7)	140 (0.6)	98 (0.8)	<.001	.14
IHD	22 (0.0)	61 (0.4)	66 (0.3)	44 (0.4)	<.001	.12
Stroke	33 (0.1)	43 (0.3)	74 (0.3)	56 (0.5)	<.001	.06
CKD[e]	446 (0.9)	211 (1.5)	263 (1.2)	215 (1.8)	<.001	<.001
Cancer	660 (1.4)	584 (4.1)	667 (3.0)	399 (3.3)	<.001	<.001
**Laboratory findings[c]**						
FBG (mg/dL)	90.4 (12.0)	108.3 (41.3)	108.8 (46.4)	107.2 (45.3)	<.001	.006
TC (mg/dL)	186.8 (35.9)	194.5 (38)	193.0 (39.5)	190.0 (40.8)	<.001	<.001
LDL-C (mg/dL)	115.6 (230.7)	119.7 (224.6)	113.7 (170.0)	107.9 (80.2)	<.001	<.001
HDL-C (mg/dL)	57.1 (24.9)	53.8 (19.9)	54.7 (21.3)	56.0 (20.9)	<.001	<.001
Triglyceride (mg/dL)	121.5 (95.1)	157.4 (129.8)	156.8 (143.7)	142.8 (131.1)	<.001	<.001
eGFR (mL/min/1.73 m^2)	99.5 (29.7)	101.2 (34.1)	103.4 (33.4)	102.9 (37.7)	<.001	<.001
**Use of antidiabetic drugs, n(%)[d]**						
Metformin	0 (0.0)	916 (6.5)	1,132 (5.1)	601 (5.0)	<.001	<.001
Sulfonylurea	0 (0.0)	914 (6.5)	1,195 (5.4)	661 (5.5)	<.001	<.001
AGI	0 (0.0)	250 (1.8)	307 (1.4)	226 (1.9)	<.001	<.001
Glinide	0 (0.0)	77 (0.5)	108 (0.5)	51 (0.4)	<.001	.40
Thiazolidinedione	0 (0.0)	168 (1.2)	123 (0.6)	65 (0.5)	<.001	<.001
DPP-4 inhibitor	0 (0.0)	9 (0.1)	14 (0.1)	5 (0.0)	<.001	.69
SGLT2 inhibitor	0 (0.0)	0 (0.0)	0 (0.0)	0 (0.0)	NA	NA
GLP1RA	0 (0.0)	0 (0.0)	0 (0.0)	0 (0.0)	NA	NA
Insulin	0 (0.0)	256 (1.8)	382 (1.7)	255 (2.1)	<.001	0.03
**Use of antihypertensive drugs, n(%)[d]**						
RAS inhibitor	632 (1.3)	1,602 (11.3)	2,024 (9.2)	974 (8.2)	<.001	<.001
Calcium channel blocker	865 (1.8)	1,524 (10.8)	2,065 (9.4)	1,104 (9.2)	<.001	<.001
Beta blocker	515 (1.1)	947 (6.7)	1,274 (5.8)	701 (5.9)	<.001	.001
Diuretics	1,502 (3.1)	1,526 (10.8)	2,246 (10.2)	1,398 (11.7)	<.001	<.001
α-blocker or vasodilator	276 (0.6)	231 (1.6)	291 (1.3)	141 (1.2)	<.001	.005
**Use of lipid-lowering drugs, n(%)[d]**						
Statin	735 (1.5)	1,744 (12.3)	1,992 (9)	988 (8.3)	<.001	<.001
Fibrate	139 (0.3)	522 (3.7)	535 (2.4)	254 (2.1)	<.001	<.001
**Use of antithrombotic drugs, n(%)[d]**	563 (1.2)	939 (6.6)	1,144 (5.2)	648 (5.4)	<.001	<.001
**No. of clinic visits per year[c]**	7.5 (6.5)	13.4 (13.1)	13.3 (11.1)	15.4 (16.5)	<.001	<.001
**No. of times participating in the national health checkup program[c]**	5.1 (3.2)	5.0 (3.4)	4.3 (2.8)	3.2 (2.3)	<.001	<.001
**(B) 40-59 years**						
**N**	294,858	117,206	103,024	80,948		
**Age (years)[c]**	50.8 (5.4)	50.7 (5.3)	51.2 (5.4)	51.2 (5.3)	<.001	<.001
**Sex, n(%)[d]**					<.001	<.001

© 2024 Kim JY et al. *JAMA Network Open*.

Men	158,510 (53.8)	69,900 (59.6)	52,854 (51.3)	36,468 (45.1)		
Women	136,348 (46.2)	47,306 (40.4)	50,170 (48.7)	44,480 (54.9)		
**BMI (kg/m^2)[c]**	23.6 (2.8)	24.8 (3.1)	24.8 (3.4)	24.7 (3.4)	<.001	<.001
**WC (cm)[c]**	79.8 (8.2)	83.5 (9.0)	83.1 (9.0)	82.6 (9.3)	<.001	<.001
**SBP (mmHg)[c]**	121.4 (14.5)	124.5 (14.7)	125.5 (15.4)	125 (15.6)	<.001	<.001
**Current smoking, n(%)[d]**	69,951 (23.7)	26,204 (22.4)	26,071 (25.3)	19,685 (24.3)	<.001	<.001
**Alcohol consumption, n(%)[d]**						
None	154,477 (52.4)	59,036 (50.4)	56,579 (54.9)	48,010 (59.3)	<.001	<.001
≤2 times/week	117,562 (39.9)	47,969 (40.9)	36,192 (35.1)	25,908 (32.0)		
≥3 times/week	20,698 (7.0)	9,487 (8.1)	9,663 (9.4)	6,671 (8.2)		
**Exercise, n(%)[d]**						
None	69,917 (23.7)	24,245 (20.7)	28,515 (27.7)	23,167 (28.6)	<.001	<.001
≤2 times/week	116,671 (39.6)	48,806 (41.6)	36,991 (35.9)	27,546 (34.0)		
≥3 times/week	106,993 (36.3)	43,713 (37.3)	37,209 (36.1)	30,000 (37.1)		
**Comorbidity, n(%)[d]**						
Hypertension	53,591 (18.2)	53,751 (45.9)	47,362 (46)	37,531 (46.4)	<.001	.08
Dyslipidemia	70,652 (24)	66,754 (57)	55,093 (53.5)	43,198 (53.4)	<.001	<.001
ASCVD	2,434 (0.8)	3,937 (3.4)	3,256 (3.2)	2,841 (3.5)	<.001	<.001
IHD	1,338 (0.5)	2,714 (2.3)	2,027 (2.0)	1,566 (1.9)	<.001	<.001
Stroke	1,125 (0.4)	1,309 (1.1)	1,293 (1.3)	1,354 (1.7)	<.001	<.001
CKD[e]	15,939 (5.4)	5,660 (4.8)	5,792 (5.6)	5,082 (6.3)	<.001	<.001
Cancer	15,030 (5.1)	11,238 (9.6)	8,460 (8.2)	6,932 (8.6)	<.001	<.001
**Laboratory findings[c]**						
FBG (mg/dL)	93.6 (12.3)	114.9 (37.7)	116.1 (41.4)	114.8 (41.2)	<.001	<.001
TC (mg/dL)	200.4 (37.6)	198.5 (41.9)	199.3 (43.8)	199 (45.2)	<.001	<.001
LDL-C (mg/dL)	120.3 (65.6)	116.1 (58.9)	115.9 (74.7)	115.6 (73.2)	<.001	.22
HDL-C (mg/dL)	56.5 (27.2)	53.4 (23.9)	54.1 (24.2)	54.6 (31.7)	<.001	<.001
Triglyceride (mg/dL)	129 (86.9)	155.9 (111.2)	158.5 (117.6)	155.6 (116.7)	<.001	<.001
eGFR (mL/min/1.73 m^2)	86 (26.1)	88.6 (29.7)	88.8 (30.1)	88.3 (30.1)	<.001	.001
**Use of antidiabetic drugs, n(%)[d]**						
Metformin	0 (0.0)	15,387 (13.1)	13,559 (13.2)	9,860 (12.2)	<.001	<.001
Sulfonylurea	0 (0.0)	17,182 (14.7)	15,692 (15.2)	11,480 (14.2)	<.001	<.001
AGI	0 (0.0)	5,675 (4.8)	5,267 (5.1)	3,946 (4.9)	<.001	.008
Glinide	0 (0.0)	1,491 (1.3)	1,268 (1.2)	953 (1.2)	<.001	.17
Thiazolidinedione	0 (0.0)	2,032 (1.7)	1,437 (1.4)	947 (1.2)	<.001	<.001
DPP-4 inhibitor	0 (0.0)	133 (0.1)	143 (0.1)	114 (0.1)	<.001	.15
SGLT2 inhibitor	0 (0.0)	0 (0.0)	0 (0.0)	0 (0.0)	NA	NA
GLP1RA	0 (0.0)	0 (0.0)	0 (0.0)	0 (0.0)	NA	NA
Insulin	0 (0.0)	3,504 (3.0)	3,323 (3.2)	2,698 (3.3)	<.001	<.001
**Use of antihypertensive drugs, n(%)[d]**						
RAS inhibitor	27,175 (9.2)	33,161 (28.3)	29,264 (28.4)	23,175 (28.6)	<.001	.26
Calcium channel blocker	34,363 (11.7)	35,431 (30.2)	33,021 (32.1)	26,117 (32.3)	<.001	<.001
Beta blocker	17,880 (6.1)	21,097 (18)	19,513 (18.9)	15,719 (19.4)	<.001	<.001
Diuretics	41,226 (14)	34,208 (29.2)	33,677 (32.7)	27,475 (33.9)	<.001	<.001
α-blocker or vasodilator	5,934 (2.0)	4,647 (4.0)	3,605 (3.5)	2,860 (3.5)	<.001	<.001
**Use of lipid-lowering drugs, n(%)[d]**						
Statin	25,289 (8.6)	34,009 (29.0)	28,457 (27.6)	22,170 (27.4)	<.001	<.001
Fibrate	3,698 (1.3)	8,230 (7.0)	6,440 (6.3)	4,690 (5.8)	<.001	<.001
**Use of antithrombotic drugs, n(%)[d]**	24,060 (8.2)	29,247 (25.0)	25,472 (24.7)	20,161 (24.9)	<.001	.44
**No. of clinic visits per year[c]**	12.3 (10.9)	20.1 (14.8)	22.1 (17.1)	24.6 (20.0)	<.001	<.001
**No. of times participating in the national health checkup program[c]**	4.8 (3.0)	4.9 (3.0)	4.3 (2.6)	4.0 (2.6)	<.001	<.001
**(C) 60-79 years**						
**N**	292,490	114,936	78,455	62,309		
**Age (years)[c]**	67.6 (5.2)	68.3 (5.2)	66.9 (5.1)	67 (5.1)	<.001	<.001
**Sex, n(%)[d]**					<.001	<.001
Men	132,563 (45.3)	50,068 (43.6)	37,148 (47.3)	28,547 (45.8)		
Women	159,927 (54.7)	64,868 (56.4)	41,307 (52.7)	33,762 (54.2)		
**BMI (kg/m^2)[c]**	23.5 (3)	24.4 (3.1)	24.4 (3.2)	24.4 (3.2)	<.001	.13
**WC (cm)[c]**	81.2 (8.5)	84.0 (9.1)	84.1 (8.5)	84.0 (8.9)	<.001	.03
**SBP (mmHg)[c]**	127.5 (15.7)	129.3 (15.7)	129.8 (16.0)	129.6 (15.9)	<.001	<.001
**Current smoking, n(%)[d]**	39,693 (13.6)	11,434 (9.9)	10,865 (13.8)	9,275 (14.9)	<.001	<.001
**Alcohol consumption, n(%)[d]**						
None	207,893 (71.1)	85,940 (74.8)	56,691 (72.3)	44,989 (72.2)	<.001	<.001
≤2 times/week	60,716 (20.8)	20,337 (17.7)	15,185 (19.4)	12,690 (20.4)		

© 2024 Kim JY et al. *JAMA Network Open*.

≥3 times/week	21,856 (7.5)	7,815 (6.8)	6,074 (7.7)	4,225 (6.8)		
**Exercise, n(%)[d]**						
None	86,314 (29.5)	32,464 (28.2)	24,262 (30.9)	19,292 (31)	<.001	<.001
≤2 times/week	80,930 (27.7)	32,035 (27.9)	21,284 (27.1)	16,842 (27)		
≥3 times/week	124,347 (42.5)	50,113 (43.6)	32,686 (41.7)	25,982 (41.7)		
**Comorbidity, n(%)[d]**						
Hypertension	133,059 (45.5)	83,927 (73.0)	55,667 (71.0)	44,577 (71.5)	<.001	<.001
Dyslipidemia	109,790 (37.5)	77,599 (67.5)	49,457 (63.0)	39,216 (62.9)	<.001	<.001
ASCVD	8,439 (2.9)	9,528 (8.3)	5,908 (7.5)	4,249 (6.8)	<.001	<.001
IHD	4,352 (1.5)	5,920 (5.2)	3,518 (4.5)	2,499 (4.0)	<.001	<.001
Stroke	4,245 (1.5)	3,975 (3.5)	2,579 (3.3)	1,898 (3.0)	<.001	<.001
CKD[e]	72,890 (24.9)	31,785 (27.7)	20,115 (25.6)	16,272 (26.1)	<.001	<.001
Cancer	29,133 (10.0)	19,283 (16.8)	11,625 (14.8)	8,738 (14.0)	<.001	<.001
**Laboratory findings[c]**						
FBG (mg/dL)	94.5 (12.5)	108.7 (29.1)	110.1 (31.6)	109.7 (31.8)	<.001	<.001
TC (mg/dL)	200.6 (39.5)	193.8 (43.3)	195.1 (41.7)	195.9 (44.1)	<.001	<.001
LDL-C (mg/dL)	121.4 (52.7)	114.3 (65.8)	114.6 (49.3)	114.9 (49.9)	<.001	.12
HDL-C (mg/dL)	55.5 (34.0)	53.6 (26.7)	53.3 (25.8)	53.8 (41.7)	<.001	.02
Triglyceride (mg/dL)	128 (74.7)	139.7 (82.4)	144.4 (88.7)	144.3 (87.9)	<.001	<.001
eGFR (mL/min/1.73 m^2)	74.4 (26.0)	73.2 (29.1)	74.2 (27.4)	73.8 (26.2)	<.001	<.001
**Use of antidiabetic drugs, n(%)[d]**						
Metformin	0 (0.0)	17,334 (15.1)	11,809 (15.1)	8,699 (14.0	<.001	<.001
Sulfonylurea	0 (0.0)	21,164 (18.4)	14,473 (18.4)	10,741 (17.2)	<.001	<.001
AGI	0 (0.0)	7,404 (6.4)	5,115 (6.5)	3,811 (6.1)	<.001	.005
Glinide	0 (0.0)	1,828 (1.6)	1,133 (1.4)	815 (1.3)	<.001	<.001
Thiazolidinedione	0 (0.0)	1,685 (1.5)	1,024 (1.3)	690 (1.1)	<.001	<.001
DPP-4 inhibitor	0 (0.0)	156 (0.1)	103 (0.1)	111 (0.2)	<.001	.04
SGLT2 inhibitor	0 (0.0)	0 (0.0)	0 (0.0)	0 (0.0)	NA	NA
GLP1RA	0 (0.0)	0 (0.0)	0 (0.0)	0 (0.0)	NA	NA
Insulin	0 (0.0)	5,930 (5.2)	3,716 (4.7)	2,787 (4.5)	<.001	<.001
**Use of antihypertensive drugs, n(%)[d]**						
RAS inhibitor	70,146 (24.0)	52,592 (45.8)	35,109 (44.8)	28,065 (45.0)	<.001	<.001
Calcium channel blocker	97,376 (33.3)	63,987 (55.7)	43,077 (54.9)	34,553 (55.5)	<.001	.004
Beta blocker	52,060 (17.8)	39,265 (34.2)	26,520 (33.8)	21,170 (34.0)	<.001	.26
Diuretics	96,546 (33.0)	60,903 (53.0)	41,436 (52.8)	33,155 (53.2)	<.001	.34
α-blocker or vasodilator	16,388 (5.6)	11,094 (9.7)	6,834 (8.7)	5,310 (8.5)	<.001	<.001
**Use of lipid-lowering drugs, n(%)[d]**						
Statin	55,340 (18.9)	46,749 (40.7)	29,454 (37.5)	23,603 (37.9)	<.001	<.001
Fibrate	6,895 (2.4)	8,448 (7.4)	5,405 (6.9)	4,185 (6.7)	<.001	<.001
**Use of antithrombotic drugs, n(%)[d]**	77,581 (26.5)	58,423 (50.8)	37,380 (47.6)	29,527 (47.4)	<.001	<.001
**No. of clinic visits per year[c]**	22.4 (18.6)	34.5 (24.3)	34.6 (26.0)	35.0 (25.7)	<.001	<.001
**No. of times participating in the national health checkup program[c]**	4.2 (2.0)	4.1 (1.7)	4.3 (1.9)	4.4 (2.3)	<.001	<.001

[a] Within controls without diabetes and individuals with type 2 diabetes.
[b] Within individuals with type 2 diabetes.
[c] Data are presented as mean (standard deviation) and compared using one-way analysis of variance (ANOVA).
[d] Data are presented as numbers (%) and compared using Pearson's chi-square test or Fisher's exact test.
[e] Defined as eGFR <60 mL/min/1.73 m^2.

AGI, alpha-glucosidase inhibitor; ASCVD, atherosclerotic cardiovascular disease; BMI, body mass index; CKD, chronic kidney disease; DPP-4, dipeptidyl peptidase-4; eGFR, estimated glomerular filtration rate; FBG, fasting blood glucose; GLP1RA, glucagon-like peptide-1 receptor agonist; HDL-C, high-density lipoprotein cholesterol; IHD, ischemic heart disease; LDL-C, low-density lipoprotein cholesterol; RAS, renin-angiotensin system; SBP, systolic blood pressure; SGLT2, sodium-glucose cotransporter 2; TC, total cholesterol; WC waist circumference.

© 2024 Kim JY et al. *JAMA Network Open*.

eTable 2. Risk of All-Cause Mortality According to Sex and Income

	No. of death (N/%)	Event rate per 1,000 person-years	OR (95% CI)		HR (95% CI)	
			Unadjusted	Adjusted[a]	Unadjusted[b]	Adjusted[a,b]
**Death from any causes**						
**Men**						
Controls without diabetes	21,006/322,358 (6.5)	7.62	ref	ref		
T2D-high income	12,610/129,345 (9.7)	10.49	1.55 (1.51–1.59)	1.47 (1.43–1.51)	ref	ref
T2D-middle income	10,621/103,742 (10.2)	11.17	1.64 (1.60–1.68)	1.80 (1.75–1.85)	1.07 (1.04–1.10)	1.19 (1.16–1.23)
T2D-low income	8,908/70,731 (12.6)	13.91	2.07 (2.01–2.12)	2.07 (2.00–2.13)	1.34 (1.30–1.37)	1.36 (1.32–1.40)
**Women**						
Controls without diabetes	10,659/313,447 (3.4)	3.98	ref	ref		
T2D-high income	7,100/116,926 (6.1)	6.46	1.84 (1.78–1.89)	1.47 (1.42–1.52)	ref	ref
T2D-middle income	5,262/99,764 (5.3)	5.64	1.58 (1.53–1.64)	1.76 (1.69–1.82)	0.88 (0.85–0.91)	1.17 (1.13–1.22)
T2D-low income	4,845/84,467 (5.7)	6.21	1.73 (1.67–1.79)	1.98 (1.91–2.06)	0.98 (0.94–1.01)	1.32 (1.28–1.37)

[a] Adjusted for age, body mass index, systolic blood pressure, smoking, drinking, exercise, underlying atherosclerotic cardiovascular disease, underlying hypertension with antihypertensive drug treatment, underlying dyslipidemia with lipid-lowering drug treatment, use of antidiabetic drugs, fasting blood glucose, low-density lipoprotein cholesterol, and estimated glomerular filtration rate.

[b] Within individuals with type 2 diabetes.

© 2024 Kim JY et al. *JAMA Network Open*.

eTable 3. Risk of Incident Atherosclerotic Cardiovascular Diseases and Cancer According to Income

	No. of events (N/%)	Event rate per 1,000 person-years	OR (95% CI)		HR (95% CI)	
			Unadjusted	Adjusted	Unadjusted[c]	Adjusted[c]
**Atherosclerotic cardiovascular disease[a]**						
Controls without diabetes	19,924/624,877 (3.2)	3.78	ref	ref		
T2D-high income	27,288/232,702 (11.7)	13.39	4.03 (3.96–4.11)	3.37 (3.31–3.44)	ref	ref
T2D-middle income	21,376/194,202 (11.0)	12.63	3.75 (3.68–3.83)	3.52 (3.45–3.60)	0.94 (0.93–0.96)	1.05 (1.03–1.07)
T2D-low income	16,552/148,010 (11.2)	12.99	3.82 (3.74–3.91)	3.52 (3.44–3.60)	0.97 (0.95–0.99)	1.06 (1.04–1.08)
**Cancer[b]**						
Controls without diabetes	52,573/590,982 (8.9)	10.78	ref	ref		
T2D-high income	31,114/215,166 (14.5)	16.46	1.73 (1.71–1.76)	1.66 (1.64–1.69)	ref	ref
T2D-middle income	24,850/182,754 (13.6)	15.55	1.61 (1.59–1.64)	1.71 (1.68–1.74)	0.95 (0.93–0.96)	1.03 (1.02–1.05)
T2D-low income	19,837/139,129 (14.3)	16.53	1.70 (1.67–1.73)	1.79 (1.76–1.82)	1.01 (0.99–1.03)	1.09 (1.07–1.11)

[a] Among subjects without atherosclerotic cardiovascular disease. Adjusted for age, sex, body mass index, systolic blood pressure, smoking, drinking, exercise, underlying hypertension with antihypertensive drug treatment, underlying dyslipidemia with lipid-lowering drug treatment, use of antidiabetic drugs, fasting blood glucose, low-density lipoprotein cholesterol, and estimated glomerular filtration rate.

[b] Among subjects without a history of cancer. Adjusted for age, sex, body mass index, systolic blood pressure, smoking, drinking, exercise, underlying atherosclerotic cardiovascular disease, underlying hypertension with antihypertensive drug treatment, underlying dyslipidemia with lipid-lowering drug treatment, use of antidiabetic drugs, fasting blood glucose, low-density lipoprotein cholesterol, and estimated glomerular filtration rate.

[c] Within individuals with type 2 diabetes.

© 2024 Kim JY et al. *JAMA Network Open*.

eTable 4. Risk of Incident Atherosclerotic Cardiovascular Diseases and Cancer According to Income and Age

	20–39 years				40–59 years				60–79 years			
	**No. of events (n/%)**	**Event rate per 1,000 person-years**	**Adjusted OR (95% CI)**	**Adjusted HR (95% CI)[c]**	**No. of events (n/%)**	**Event rate per 1,000 person-years**	**Adjusted OR (95% CI)**	**Adjusted HR (95% CI)[c]**	**No. of events (n/%)**	**Event rate per 1,000 person-years**	**Adjusted OR (95% CI)**	**Adjusted HR (95% CI)[c]**
**Atherosclerotic cardiovascular disease[a]**												
Controls without diabetes	157/48,402 (0.3)	0.36	ref		5,365/292,424 (1.8)	2.12	ref		14,402/284,051 (5.1)	6.24	ref	
T2D-high income	330/14,025 (2.4)	2.52	4.77 (3.92–5.82)	ref	9,291/113,269 (8.2)	9.09	3.88 (3.74–4.03)	ref	17,667/105,408 (16.8)	19.95	3.17 (3.09–3.25)	ref
T2D-middle income	603/21,887 (2.8)	3.04	6.66 (5.54–8.00)	1.40 (1.22–1.61)	8,572/99,768 (8.6)	9.63	4.03 (3.88–4.18)	1.05 (1.02–1.08)	12,201/72,547 (16.8)	20.19	3.30 (3.21–3.39)	1.05 (1.02–1.07)
T2D-low income	292/11,843 (2.5)	2.69	6.76 (5.52–8.28)	1.41 (1.20–1.66)	6,814/78,107 (8.7)	9.89	4.23 (4.06–4.39)	1.10 (1.07–1.14)	9,446/58,060 (16.3)	19.80	3.17 (3.08–3.27)	1.02 (1.00–1.05)
P-values comparing the odds ratio of the T2D-low income group over the controls without diabetes in age 20–39 years vs. that in age 60–79 years: <.001												
P-values comparing the risk ratio of the T2D-low income group over the T2D-high income group in age 20–39 years vs. that in age 60–79 years[c]: .03												
**Cancer[b]**												
Controls without diabetes	1,199/47,797 (2.5)	2.83	ref		18,566/279,828 (6.6)	7.84	ref		32,808/263,357 (12.5)	15.76	ref	
T2D-high income	816/13,545 (6.0)	6.55	2.13 (1.93–2.34)	ref	11,977/105,968 (11.3)	12.58	1.73 (1.68–1.77)	ref	18,321/95,653 (19.2)	22.51	1.62 (1.59–1.66)	ref
T2D-middle income	1,049/21,360 (4.9)	5.46	1.87 (1.71–2.04)	0.91 (0.83–1.00)	11,088/94,564 (11.7)	13.19	1.76 (1.71–1.81)	1.03 (1.003–1.06)	12,713/66,830 (19.0)	22.51	1.61 (1.57–1.65)	1.01 (0.98–1.03)
T2D-low income	652/11,542 (5.6)	6.26	1.95 (1.76–2.16)	0.96 (0.87–1.07)	8,879/74,016 (12.0)	13.65	1.81 (1.76–1.87)	1.07 (1.04–1.10)	10,306/53,571 (19.2)	23.14	1.65 (1.61–1.69)	1.04 (1.01–1.06)
P-values comparing the odds ratio of the T2D-low income group over the controls without diabetes in age 20–39 years vs. that in age 60–79 years: <.001												
P-values comparing the risk ratio of the T2D-low income group over the T2D-high income group in age 20–39 years vs. that in age 60–79 years[c]: .83												

ORs and HRs were calculated within each age group.

[a] Among subjects without atherosclerotic cardiovascular disease. Adjusted for age, sex, body mass index, systolic blood pressure, smoking, drinking, exercise, underlying hypertension with antihypertensive drug treatment, underlying dyslipidemia with lipid-lowering drug treatment, use of antidiabetic drugs, fasting blood glucose, low-density lipoprotein cholesterol, and estimated glomerular filtration rate.

[b] Among subjects without a history of cancer. Adjusted for age, sex, body mass index, systolic blood pressure, smoking, drinking, exercise, underlying atherosclerotic cardiovascular disease, underlying hypertension with antihypertensive drug treatment, underlying dyslipidemia with lipid-lowering drug treatment, use of antidiabetic drugs, fasting blood glucose, low-density lipoprotein cholesterol, and estimated glomerular filtration rate.

[c] Within subjects with type 2 diabetes

© 2024 Kim JY et al. *JAMA Network Open*.

eTable 5. Multivariable Analysis of All-Cause Mortality in Individuals With Type 2 Diabetes Aged 20 to 39 Years

	**HR (95% CI)**
Income status	
T2D-high income	ref
T2D-middle income	1.74 (1.37–2.21)
T2D-low income	2.88 (2.25–3.69)
Age (years)	1.06 (1.04–1.08)
Female sex (male as a reference)	0.64 (0.5–0.81)
BMI (kg/m^2)	0.91 (0.89–0.93)
SBP (mmHg)	1.01 (1.003–1.02)
Smoking	
None	ref
Current	1.83 (1.50–2.24)
Unknown	1.04 (0.12–8.86)
Drinking	
None	ref
≤2 times/week	0.86 (0.71–1.05)
≥3 times/week	1.35 (0.99–1.84)
Unknown	0.78 (0.15–3.95)
Regular exercise	
None	ref
≤2 times/week	0.77 (0.62–0.96)
≥3 times/week	0.84 (0.68–1.05)
Unknown	1.10 (0.22–5.46)
ASCVD	1.55 (0.84–2.87)
HTN	2.15 (1.77–2.60)
Dyslipidemia	1.07 (0.83–1.37)

Hazard ratios (HRs) and 95% confidence intervals (CIs) were calculated using Cox proportional hazard regression models.

ASCVD, atherosclerotic cardiovascular disease; BMI, body mass index; eGFR, estimated glomerular filtration rate; HTN, hypertension; LDL, low-density lipoprotein; SBP, systolic blood pressure.

© 2024 Kim JY et al. *JAMA Network Open*.

eTable 6. Causes of Death in the Total Population

	N	%	Ranking
**Total population**			
Neoplasm (C00-D48)	31340	38.9%	1
Diseases of the circulatory system (I00-I99)	15402	19.1%	2
External causes of morbidity and mortality (V01-Y98)	9984	12.4%	3
Diseases of the respiratory system (J00-J99)	6957	8.6%	4
Symptoms, signs and abnormal clinical and laboratory findings, NEC (R00-R99)	5196	6.4%	5
Diseases of the digestive system (K00-K93)	2977	3.7%	6
Diseases of the nervous system (G00-G99)	2297	2.8%	7
Certain infectious and parasitic diseases (A00-B99)	1893	2.3%	8
Endocrine, nutritional and metabolic diseases (E00-E90)	1833	2.3%	9
Diseases of the genitourinary system (N00-N99)	984	1.2%	10
Mental and behavioural disorders (F00-F99)	833	1.0%	11
Diseases of the musculoskeletal system and connective tissue (M00-M99)	360	0.4%	12
Congenital malformations, deformations and chromosomal abnormalities (Q00-Q99)	250	0.3%	13
Diseases of the blood and blood-forming organs and certain disorders involving the immune mechanism (D50-D89)	193	0.2%	14
Diseases of the skin and subcutaneous tissue (L00-L99)	97	0.1%	15
Pregnancy, childbirth and the puerperium (O00-O99)	6	0.0%	16
Total	80602	100.0%	

© 2024 Kim JY et al. *JAMA Network Open*.

eTable 7. Causes of Death by Age and Income

(A) 20–39 years

	Controls without diabetes			T2D-high income			T2D-middle income			T2D-low income		
	N	%	rank	N	%	rank	N	%	rank	N	%	rank
**Neoplasm (C00-D48)**	**32**	**16.8%**	**2**	**37**	**38.9%**	**1**	**67**	**27.8%**	**2**	**40**	**19.6%**	**2**
**Diseases of the circulatory system (I00-I99)**	**26**	**13.6%**	**3**	**12**	**12.6%**	**3**	**37**	**15.4%**	**3**	**23**	**11.3%**	**4**
**External causes of morbidity and mortality (V01-Y98)**	**111**	**58.1%**	**1**	**26**	**27.4%**	**2**	**72**	**29.9%**	**1**	**63**	**30.9%**	**1**
Diseases of the respiratory system (J00-J99)	1	0.5%	7	1	1.1%	10	4	1.7%	9	4	2.0%	7
Symptoms, signs and abnormal clinical and laboratory findings, NEC (R00-R99)	13	6.8%	4	3	3.2%	5	10	4.1%	5	18	8.8%	5
Diseases of the digestive system (K00-K93)	2	1.0%	5	6	6.3%	4	27	11.2%	4	35	17.2%	3
Diseases of the nervous system (G00-G99)	1	0.5%	7	2	2.1%	6	5	2.1%	7	2	1.0%	9
Certain infectious and parasitic diseases (A00-B99)	2	1.0%	5	0	0.0%	13	6	2.5%	6	4	2.0%	7
Endocrine, nutritional and metabolic diseases (E00-E90)	0	0.0%	12	1	1.1%	10	5	2.1%	7	7	3.4%	6
Diseases of the genitourinary system (N00-N99)	1	0.5%	7	1	1.1%	10	0	0.0%	14	2	1.0%	9
Mental and behavioural disorders (F00-F99)	0	0.0%	12	2	2.1%	6	2	0.8%	11	2	1.0%	9
Diseases of the musculoskeletal system and connective tissue (M00-M99)	0	0.0%	12	0	0.0%	13	3	1.2%	10	0	0.0%	15
Congenital malformations, deformations and chromosomal abnormalities (Q00-Q99)	0	0.0%	12	2	2.1%	6	0	0.0%	14	1	0.5%	13
Diseases of the blood and blood-forming organs and certain disorders involving the immune mechanism (D50-D89)	1	0.5%	7	0	0.0%	13	2	0.8%	11	0	0.0%	15
Diseases of the skin and subcutaneous tissue (L00-L99)	0	0.0%	12	0	0.0%	13	0	0.0%	14	1	0.5%	13
Pregnancy, childbirth and the puerperium (O00-O99)	1	0.5%	7	2	2.1%	6	1	0.4%	13	2	1.0%	9
Total	191	100.0%		95	100.0%		241	100.0%		204	100.0%	

© 2024 Kim JY et al. *JAMA Network Open*.

(B) 40–59 years	Controls without diabetes			T2D-high income			T2D-middle income			T2D-low income		
	N	%	rank	N	%	rank	N	%	rank	N	%	rank
**Neoplasm (C00-D48)**	**1869**	**40.5%**	**1**	**1425**	**49.1%**	**1**	**1601**	**41.0%**	**1**	**1332**	**35.0%**	**1**
**Diseases of the circulatory system (I00-I99)**	**628**	**13.6%**	**3**	**400**	**13.8%**	**3**	**583**	**14.9%**	**3**	**621**	**16.3%**	**2**
**External causes of morbidity and mortality (V01-Y98)**	**1430**	**31.0%**	**2**	**538**	**18.5%**	**2**	**630**	**16.1%**	**2**	**600**	**15.7%**	**3**
Diseases of the respiratory system (J00-J99)	82	1.8%	6	63	2.2%	7	163	4.2%	6	181	4.8%	6
Symptoms, signs and abnormal clinical and laboratory findings, NEC (R00-R99)	272	5.9%	4	88	3.0%	5	178	4.6%	5	257	6.7%	5
Diseases of the digestive system (K00-K93)	148	3.2%	5	155	5.3%	4	326	8.4%	4	361	9.5%	4
Diseases of the nervous system (G00-G99)	59	1.3%	7	52	1.8%	8	65	1.7%	9	69	1.8%	9
Certain infectious and parasitic diseases (A00-B99)	41	0.9%	9	44	1.5%	9	93	2.4%	8	92	2.4%	8
Endocrine, nutritional and metabolic diseases (E00-E90)	17	0.4%	10	76	2.6%	6	149	3.8%	7	148	3.9%	7
Diseases of the genitourinary system (N00-N99)	8	0.2%	12	27	0.9%	10	50	1.3%	10	64	1.7%	10
Mental and behavioural disorders (F00-F99)	47	1.0%	8	11	0.4%	12	35	0.9%	11	46	1.2%	11
Diseases of the musculoskeletal system and connective tissue (M00-M99)	8	0.2%	12	13	0.4%	11	15	0.4%	12	27	0.7%	12
Congenital malformations, deformations and chromosomal abnormalities (Q00-Q99)	0	0.0%	14	2	0.1%	14	6	0.2%	14	1	0.0%	15
Diseases of the blood and blood-forming organs and certain disorders involving the immune mechanism (D50-D89)	9	0.2%	11	9	0.3%	13	7	0.2%	13	7	0.2%	13
Diseases of the skin and subcutaneous tissue (L00-L99)	0	0.0%	14	0	0.0%	15	1	0.0%	15	4	0.1%	14
Pregnancy, childbirth and the puerperium (O00-O99)	0	0.0%	14	0	0.0%	15	0	0.0%	16	0	0.0%	16
Total	4618	100.0%		2903	100.0%		3902	100.0%		3810	100.0%	

© 2024 Kim JY et al. *JAMA Network Open*.

(A) 60–79 years												
	Controls without diabetes			T2D-high income			T2D-middle income			T2D-low income		
	N	%	rank	N	%	rank	N	%	rank	N	%	rank
**Neoplasm (C00-D48)**	**10891**	**40.8%**	**1**	**6177**	**37.1%**	**1**	**4305**	**36.9%**	**1**	**3564**	**36.9%**	**1**
**Diseases of the circulatory system (I00-I99)**	**4969**	**18.6%**	**2**	**3526**	**21.2%**	**2**	**2495**	**21.4%**	**2**	**2082**	**21.6%**	**2**
**External causes of morbidity and mortality (V01-Y98)**	**3517**	**13.2%**	**3**	**1250**	**7.5%**	**4**	**982**	**8.4%**	**4**	**765**	**7.9%**	**4**
Diseases of the respiratory system (J00-J99)	2297	8.6%	4	1897	11.4%	3	1243	10.7%	3	1021	10.6%	3
Symptoms, signs and abnormal clinical and laboratory findings, NEC (R00-R99)	2163	8.1%	5	953	5.7%	5	654	5.6%	5	587	6.1%	5
Diseases of the digestive system (K00-K93)	620	2.3%	7	526	3.2%	8	418	3.6%	7	353	3.7%	7
Diseases of the nervous system (G00-G99)	841	3.2%	6	579	3.5%	6	343	2.9%	8	279	2.9%	8
Certain infectious and parasitic diseases (A00-B99)	559	2.1%	8	487	2.9%	9	324	2.8%	9	241	2.5%	9
Endocrine, nutritional and metabolic diseases (E00-E90)	100	0.4%	11	532	3.2%	7	434	3.7%	6	364	3.8%	6
Diseases of the genitourinary system (N00-N99)	192	0.7%	10	395	2.4%	10	244	2.1%	10	0	0.0%	15
Mental and behavioural disorders (F00-F99)	344	1.3%	9	152	0.9%	11	114	1.0%	11	78	0.8%	11
Diseases of the musculoskeletal system and connective tissue (M00-M99)	94	0.4%	12	95	0.6%	12	60	0.5%	12	45	0.5%	12
Congenital malformations, deformations and chromosomal abnormalities (Q00-Q99)	2	0.0%	15	6	0.0%	15	5	0.0%	15	225	2.3%	10
Diseases of the blood and blood-forming organs and certain disorders involving the immune mechanism (D50-D89)	57	0.2%	13	45	0.3%	13	31	0.3%	13	25	0.3%	13
Diseases of the skin and subcutaneous tissue (L00-L99)	36	0.1%	14	21	0.1%	14	13	0.1%	14	21	0.2%	14
Pregnancy, childbirth and the puerperium (O00-O99)	0	0.0%	16	0	0.0%	16	0	0.0%	16	0	0.0%	15
Total	26682	100.0%		16641	100.0%		11665	100.0%		9650	100.0%	

© 2024 Kim JY et al. *JAMA Network Open*.

eTable 8. Analysis of the Risk of Overall and Cause-Specific Mortality by Income and Age, Using Different *ICD-10* Codes for T2D

	20–39 years				40–59 years				60–79 years			
	No. of subjects	No. of events	Adjusted OR (95% CI)[a,b]	Adjusted HR (95% CI)[b,c]	No. of subjects	No. of events	Adjusted OR (95% CI)[a,b]	Adjusted HR (95% CI)[b,c]	No. of subjects	No. of events	Adjusted OR (95% CI)[a,b]	Adjusted HR (95% CI)[b,c]
**Among individuals receiving E11 code**												
**Death from any causes**												
Controls without diabetes	48,457	193	ref		294,858	4,655	ref		292,490	26,817	ref	
T2D-high income	9,641	61	1.24 (0.92–1.68)	ref	84,315	2,101	1.40 (1.32–1.48)	ref	83,114	12,266	1.44 (1.40–1.48)	ref
T2D-middle income	14,818	168	2.36 (1.89–2.95)	1.93 (1.44–2.60)	75,171	2,899	2.08 (1.98–2.20)	1.49 (1.41–1.57)	57,173	8,569	1.69 (1.64–1.74)	1.16 (1.13–1.19)
T2D-low income	8,084	129	3.58 (2.81–4.56)	2.88 (2.11–3.93)	58,638	2,839	2.74 (2.59–2.89)	1.94 (1.83–2.05)	45,389	7,114	1.82 (1.76–1.88)	1.25 (1.22–1.29)
**Death from cardiovascular disease**												
Controls without diabetes	48,457	26	ref		294,858	628	ref		292,490	4,969	ref	
T2D-high income	9,641	8	0.82 (0.35–1.91)	ref	84,315	293	1.16 (1.00–1.35)	ref	83,114	2,592	1.31 (1.24–1.38)	ref
T2D-middle income	14,818	25	1.87 (1.02–3.45)	2.20 (0.98–4.93)	75,171	445	1.85 (1.61–2.12)	1.62 (1.39–1.87)	57,173	1,792	1.52 (1.43–1.62)	1.17 (1.10–1.25)
T2D-low income	8,084	16	2.49 (1.25–4.95)	2.93 (1.23–6.97)	58,638	474	2.56 (2.23–2.92)	2.26 (1.95–2.62)	45,389	1,542	1.68 (1.58–1.79)	1.32 (1.23–1.40)
**Death from cancer**												
Controls without diabetes	48,457	32	ref		294,858	1,856	ref		292,490	10,737	ref	
T2D-high income	9,641	19	2.26 (1.25–4.10)	ref	84,315	986	1.82 (1.67–1.98)	ref	83,114	4,386	1.37 (1.32–1.43)	ref
T2D-middle income	14,818	44	3.78 (2.33–6.13)	1.66 (0.96–2.86)	75,171	1,149	2.26 (2.09–2.46)	1.27 (1.16–1.38)	57,173	3,086	1.53 (1.46–1.60)	1.12 (1.07–1.17)
T2D-low income	8,084	22	3.41 (1.92–6.05)	1.52 (0.81–2.85)	58,638	974	2.52 (2.32–2.75)	1.43 (1.31–1.56)	45,389	2,578	1.63 (1.55–1.71)	1.21 (1.15–1.27)
**Among individuals receiving E12–14 codes**												
**Death from any causes**												
Controls without diabetes	48,457	193	ref		294,858	4,655	ref		292,490	26,817	ref	

© 2024 Kim JY et al. *JAMA Network Open*.

T2D-high income	4,488	35	1.67 (1.15–2.44)	ref	32,891	811	1.45 (1.34–1.57)	ref	31,822	4,436	1.38 (1.33–1.44)	ref
T2D-middle income	7,209	74	2.28 (1.72–3.03)	1.39 (0.92–2.08)	27,853	1,032	2.08 (1.93–2.24)	1.44 (1.32–1.59)	21,282	3,141	1.68 (1.61–1.76)	1.19 (1.14–1.25)
T2D-low income	3,857	77	4.85 (3.66–6.44)	2.81 (1.87–4.24)	22,310	1,004	2.62 (2.43–2.83)	1.80 (1.64–1.98)	16,920	2,590	1.78 (1.70–1.87)	1.28 (1.22–1.35)
**Death from cardiovascular disease**												
Controls without diabetes	48,457	26	ref		294,858	628	ref		292,490	4,969	ref	
T2D-high income	4,488	4	0.89 (0.29–2.68)	ref	32,891	107	1.17 (0.94–1.46)	ref	31,822	934	1.26 (1.17–1.36)	ref
T2D-middle income	7,209	12	1.89 (0.89–4.01)	2.09 (0.66–6.67)	27,853	138	1.63 (1.33–1.99)	1.40 (1.09–1.81)	21,282	703	1.63 (1.50–1.78)	1.31 (1.19–1.45)
T2D-low income	3,857	7	2.34 (0.96–5.70)	2.54 (0.72–9.01)	22,310	147	2.16 (1.77–2.63)	1.87 (1.45–2.40)	16,920	540	1.59 (1.44–1.75)	1.32 (1.18–1.47)
**Death from cancer**												
Controls without diabetes	48,457	32	ref		294,858	1,856	ref		292,490	10,737	ref	
T2D-high income	4,488	17	5.12 (2.77–9.45)	ref	32,891	421	2.02 (1.81–2.27)	ref	31,822	1,666	1.37 (1.30–1.45)	ref
T2D-middle income	7,209	20	4.20 (2.35–7.50)	0.83 (0.43–1.60)	27,853	427	2.31 (2.06–2.59)	1.18 (1.03–1.36)	21,282	1,155	1.52 (1.42–1.62)	1.10 (1.02–1.19)
T2D-low income	3,857	17	6.81 (3.68–12.60)	1.36 (0.68–2.71)	22,310	343	2.36 (2.09–2.68)	1.22 (1.06–1.41)	16,920	930	1.56 (1.45–1.67)	1.15 (1.06–1.25)

[a] ORs were calculated from the models fitted in individual age ranges, including controls without diabetes and individuals with type 2 diabetes.

[b] Adjusted for age, sex, body mass index, systolic blood pressure, smoking, drinking, exercise, underlying atherosclerotic cardiovascular disease, underlying hypertension with antihypertensive drug treatment, underlying dyslipidemia with lipid-lowering drug treatment, use of antidiabetic drugs, fasting blood glucose, low-density lipoprotein cholesterol, and estimated glomerular filtration rate.

[c] Within individuals with type 2 diabetes. HRs were calculated from the models fitted in individual age ranges.

© 2024 Kim JY et al. *JAMA Network Open*.

eFigure. Mortality Rate According to Income and Age

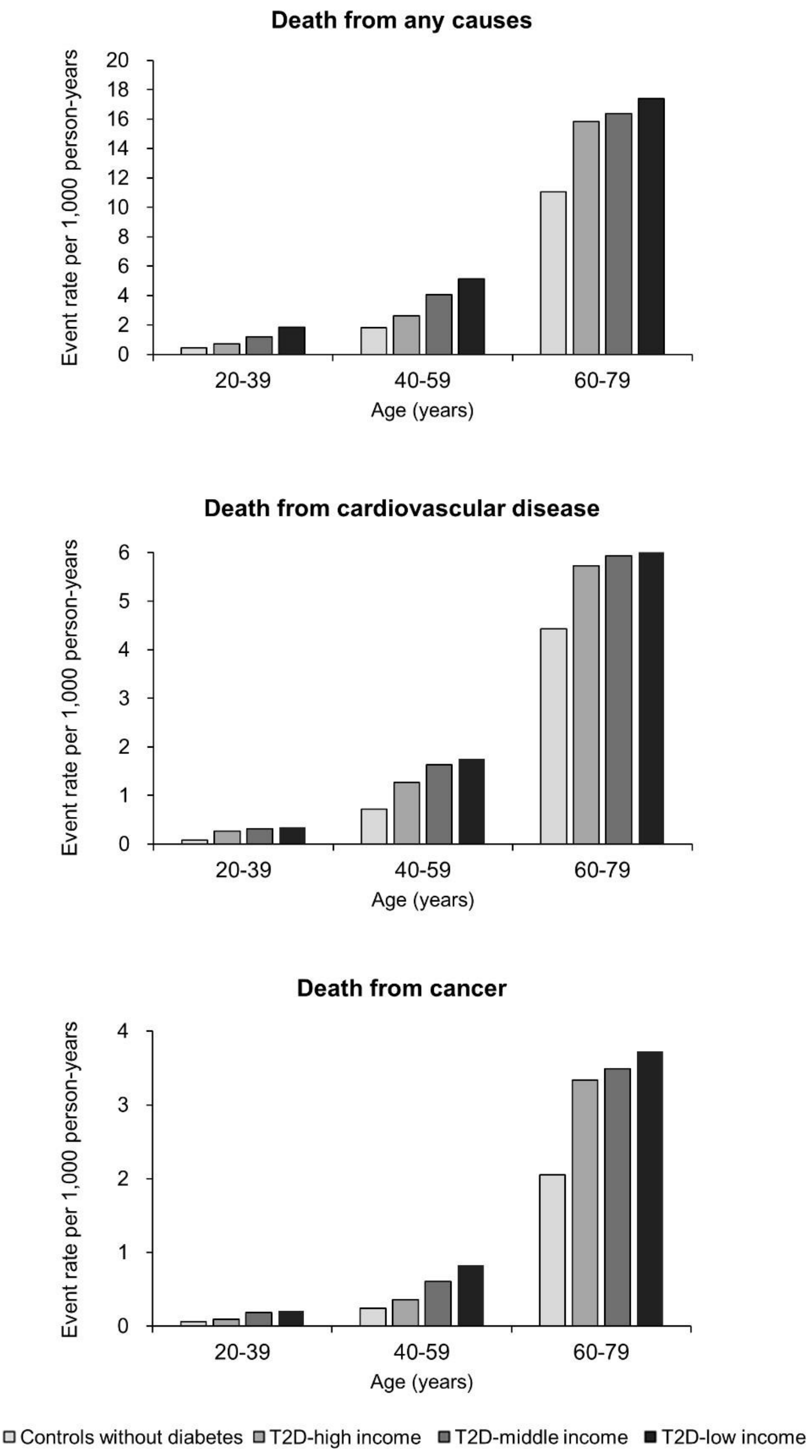

© 2024 Kim JY et al. *JAMA Network Open*.
