# Supplementary material for: Income-Related Disparities in Mortality Among Young Adults With Type 2 Diabetes
Source: JAMA Netw Open. 2024 Nov 12;7(11):e2443918. doi: 10.1001/jamanetworkopen.2024.43918 (PMC11558478; doi:10.1001/jamanetworkopen.2024.43918)
Supplement: Supplement 2. — Data Sharing Statement [file jamanetwopen-e2443918-s002.pdf]

Data Sharing Statement

Kim. Income-Related Disparities in Mortality Among Young Adults With Type 2 Diabetes. *JAMA Netw Open*. Published November 12, 2024. doi:10.1001/jamanetworkopen.2024.43918

Data
Data available: No

Additional Information
Explanation for why data not available: Additional data are available through approval and oversight by the Korean National Health Insurance Service (available at https://nhiss.nhis.or.kr).
